# Myricetin Inhibits SARS-CoV-2 Viral Replication by Targeting M^pro^ and Ameliorates Pulmonary Inflammation

**DOI:** 10.3389/fphar.2021.669642

**Published:** 2021-06-17

**Authors:** Ting Xiao, Mengqi Cui, Caijuan Zheng, Ming Wang, Ronghao Sun, Dandi Gao, Jiali Bao, Shanfa Ren, Bo Yang, Jianping Lin, Xiaoping Li, Dongmei Li, Cheng Yang, Honggang Zhou

**Affiliations:** ^1^ State Key Laboratory of Medicinal Chemical Biology, College of Pharmacy and Tianjin Key Laboratory of Molecular Drug Research, Nankai University, Haihe Education Park, Tianjin, China; ^2^ Tianjin Key Laboratory of Molecular Drug Research, Tianjin International Joint Academy of Biomedicine, Tianjin, China; ^3^ Department of Thoracic Surgery, Tianjin First Central Hospital, Nankai University, Tianjin, China

**Keywords:** COVID-19, SARS-CoV-2, 3CLpro (Mpro), myricetin, pulmonary inflammation

## Abstract

The coronavirus disease 2019 (COVID-19) has spread widely around the world and has seriously affected the human health of tens of millions of people. In view of lacking anti-virus drugs target to SARS-CoV-2, there is an urgent need to develop effective new drugs. In this study, we reported our discovery of SARS-CoV-2 M^pro^ inhibitors. We selected 15 natural compounds, including 7 flavonoids, 3 coumarins, 2 terpenoids, one henolic, one aldehyde and one steroid compound for molecular docking and enzymatic screening. Myricetin were identified to have potent inhibit activity with IC_50_ 3.684 ± 0.076 μM in the enzyme assay. The binding pose of Myricetin with SARS-CoV-2 M^pro^ was identified using molecular docking method. In the binding pocket of SARS-CoV-2 M^pro^, the chromone ring of Myricetin interacts with His41 through *π*-π stacking, and the 3’-, 4’- and 7-hydroxyl of Myricetin interact with Phe140, Glu166and Asp187 through hydrogen bonds. Significantly, our results showed that Myricetin has potent effect on bleomycin-induced pulmonary inflammation by inhibiting the infiltration of inflammatory cells and the secretion of inflammatory cytokines IL-6, IL-1α, TNF-α and IFN-γ. Overall, Myricetin may be a potential drug for anti-virus and symptomatic treatment of COVID-19.

## Introduction

The new type of coronavirus pneumonia is called COVID-19, which is a viral respiratory disease caused by the SARS-CoV-2 infection (Mittal et al., 2020; Wang et al., 2020). COVID-19 caused a global health emergency and was declared a pandemic by the World Health Organization (Ciotti et al., 2019; Kumar et al., 2020). The spread of COVID-19 brought great harm and social impact (Tandon, 2020). As of December 1, 2020, the cumulative number of confirmed cases has near to 70 millions all over the world. The overall mortality reaches about 2.19% (Cucinotta and Vanelli, 2020). Based on the data from the Chinese National Reporting System, as of February 20, 2020, 80% of the reported confirmed cases were without pneumonie, or had mild to mode rate pneumonia; about 15% had severe pneumonia (Park, 2020). Although some mild patients can heal on their own, there are still many patients who progress rapidly in the later stages, and develop into acute respiratory distress syndrome and fibrosis (Ozma et al., 2020). And now, there are still no specific medicines and effective therapeutic methods. Therefore, there is an urgent need to developing specific drugs for COVID-19.

SARS-CoV-2 is a single positive-stranded RNA virus, it contains about 30,000 basic group and 14 open reading frames (ORFs), which can coding replicases, four structural proteins (Spike, Envelope, Membrane and Nucleocapsid protein), 16 non-structural proteins (NSPs) and nine accessory proteins (Boopathi et al., 2020; Mittal et al., 2020; Sarma et al., 2020). NSPs play an important role in the replication and transcription cycle of the virus. NSP5 is the main protease of SARS-CoV-2, also called 3CLpro, it is essential for viral polyproteins processing and maturation (Anand et al., 2003; Jin et al., 2020; Liu et al., 2020; Zhang et al., 2020), therefore, it is recognized as an important potential drug target (De Clercq and Li, 2016).

Natural products (NPs) received great attention by scientific to discover potential drugs for the treatment of various diseases, such as cancer (Cragg et al., 1997), HIV (Kurapati et al., 2015), malaria (Clark, 1996) and cardiovascular disease (Mashour et al., 1998). And recently, a large of studies reported the screening result of NPs as anti-SARS-CoV-2 inhibitors based on *in-silico* drug discovery approaches (Ibrahim et al., 2020; Joshi et al., 2020; Li et al., 2020), but there are few reports on the directly inhibition of enzyme activity. We identified Myricetin as a potent inhibitor of SARS-CoV-2 M^pro^ from 15 NPs by molecular docking and enzymatic assay in this study. Myricetin also exhibit potent anti-inflammation effect on bleomycin-treated mice. It suggests that Myricetin might be a promising candidate for COVID-19 therapy.

## Method

### Drugs and Reagents

The 15 test compounds were mainly obtained from Pusi Biotechnology Co. Ltd. (Chengdu, China). The enzyme activity inhibitor screening kit was purchased from Beyotime Biotechnology (Shanghai, China).

### Molecular Docking

The crystal structure (PDB ID: 6LZE) of SARS-CoV-2 M^pro^, which was resolved by Dai et al. (Dai et al., 2020), was extracted from the RCSB Protein Data Bank (PDB). Then, the protein structure was prepared using the Protein Preparation Wizard module in Schrodinger 2017 (Bhachoo and Beuming, 2017) to remove all crystallographic water molecules, correct side chains with missing atoms, add hydrogen atoms and assign protonation states and partial charges with the OPLS_2005 force field. The Protein Preparation Wizard module of Schrödinger was applied to add hydrogen. The protonation states for the hydroxyl, Asn, Gln, and His were optimized using the ProtAssign module of Schrödinger. After that, the protein structure was minimized until the root-mean-square deviation (RMSD) of the nonhydrogen atoms reached less than 0.3 Å. The structures of the 15 natural compounds and 17 chemical compounds were prepared using the LigPrep module of the Schrodinger 2017 molecular modeling package to add hydrogen atoms, convert 2D structures to 3D, generate stereoisomers and determine the ionization state at pH 7.0 ± 2.0 with Epik. Using the prepared receptor structure, a receptor grid was generated around the original ligand site of the crystal structure. Then, the 15 natural compounds and 17 chemical compounds were docked to the receptor using the Glide XP protocol.

### Protease Activity Assay

Enzyme activity inhibitor screening adopts fluorescence resonance energy transfer method. The protease assays were performed in 96-well black flat-bottomed plates with a final volume of 100 μl. The SARS-CoV-2 M^pro^, at a final concentration of 0.3 μM was pre-incubated for 5 min at 37°C with different compounds, at a final concentration of 50 μM in the assay buffer (50 mM Tris, 150 mM NaCl, 1 mM EDTA, 1%glycerol, PH7.3). The FRET substrate, Dabcyl-KTSAVLQSGFRKME-Edans (Jin et al., 2020), is added at a final concentration of 20 μM to the enzymatic reaction mixture for 10 min at 37°C. The blank control well consists of 93 μl assay buffer, 5 μl DMSO and 2 μl Substrate. Enzyme activity control well contains of 92 μl assay buffer, 1 μl M^pro^, 5 μl DMSO and 2 μl Substrate, sample wells are 92 μl assay buffer, 1 μl M^pro^, 5 μl compound and 2 μl Substrate. After incubating at 37°C for 5 min in the dark, the fluorescence signals (excitation/emission, 340 nm/490 nm) of released EDANS were measured using a multiscan spectrum (Thermo, United States). The results were plotted as dose inhibition curves using nonlinear regression with a variable slope to determine the IC_50_ values by GraghPad Prism 7.0.

### Molecular Dynamics Simulation

To investigate the stability of Myricetin inside the active site of SARS-CoV-2 M^pro^, molecular dynamics (MD) simulation was performed on the binding complex of SARS-CoV-2 M^pro^ with Myricetin obtained from the molecular docking. The MD simulation was carried out using the PMEMD module of AMBER18. The AMBER FF14SB force field (Maier et al., 2015) was used for SARS-CoV-2 M^pro^ and the GAFF force field (Wang et al., 2004) was used for Myricetin. The binding complex was neutralized by adding sodium counterions and was solvated in a rectangular box of TIP3P water molecules, with a minimal distance of 12 Å from the protein to the box boundary. The system was subject to energy minimization for 10,000 steps. Next, the complex was gradually heated from 0 to 310 K, followed by equilibration for 5 ns using NVT ensemble, and the protein and ligand were constrained with a force constraint of 50 kcal mol^−1^·Å^−2^. Then, the system was equilibrated for 30 ns using the NPT ensemble with constraint force constant gradually decreased and finally removed for the production MD simulation. The production MD at 310 K was kept running 100 ns to obtain a stable MD trajectory. During the MD simulation, a 12 Å nonbonded interaction cutoff was used, the SHAKE algorithm integration was used to constrain covalent bonds that involved hydrogen atoms and the particle mesh Ewald (PME) method was applied to treat long-range electrostatic interactions. The frames were saved every 5000 steps for analysis. Binding free energy between the SARS-CoV-2 M^pro^ and Myricetin was calculated with the MM-GBSA method.

### Cytotoxicity Assay

BEAS-2B cells were cultured at 37°C with 5% CO2 in a humid atmosphere. BEAS-2B cells were maintained in 96-well plates at 5× 10^4^ cells/ml, and were cultured with serially twice diluted Myricetin for 48 h. 15 μl MTT reagent was added in each well of 96-well plate, Cell viability was measured after 4 h of culture at 37°C. The resulting formazan crystals were dissolved with 120 μl of DMSO solution. The value of OD was measured at a wavelength of 570 nm by using Thermo Scientific™ Multiskan™ FC (New York, NY, United States). These experimental results were repeated at least three times.

### Animals and Bleomycin Administration

Male C57BL/six mice (6–8 weeks, 20–25 g) were purchased from Charles River Laboratory (Beijing, China). All animal feeding and testing procedures comply with the criteria approved by the Institutional Animal Care and Use Committee (IACUC) of Nankai University (Permit No. SYXK 2014-0003). Mice were exposed to a controlled temperature (22–26°C), humidity (60 ± 2%)and a 12 h cycle of light and dark, giving them free access to food and water.

Mice were intratracheal injected with bleomycin (BLM). In short, mice were anesthetized by intraperitoneal injection of 1% pentobarbital sodium, followed by intratracheal injection of 2.5 U/kg bleomycin (BLM, China Hanfai Manufacturing Co., LTD.) with sterile insulin syringe. After injection, the mice were immediately raised and gently flapped to evenly distribute the liquid in the lungs. In the control group, the same method was used to inject the same amount of normal saline (0.9% NaCl), the 30 mice were randomly divided into six groups, with five mice in each group: control group, BLM model group, BLM + pirfenidone (PFD) group (200 mg/kg), BLM + Myricetin group (25 mg/kg), BLM + Myricetin group (50 mg/kg), BLM + Myricetin group (100 mg/kg). Pirfenidone was used as positive control. The drug Pirfenidone or Myricetin was given daily intragastric administration 1–7 days after BLM injury, the control group and BLM model group were given the same amount of normal saline. Mice were euthanized on the eighth day after administration to assess pulmonary inflammation.

### Bronchoalveolar Lavage Fluid

The lungs were laved with PBS to collect bronchoalveolar lavage fluid (BALF), underwent lavage through a blunt needle attached to a syringe, which worked as a trachea cannula in the airway. Bronchoalveolar lavage fluid (BALF) was collected by washing the lung through a tracheal intubation. The lungs were washed twice times, and each time 1 ml PBS was used,rinse once and twice for the second time. The BALF was centrifuged at 3,000 rpm for 10 min and collected the supernatant and stored at −80°C. The supernatant was used for inflammatory factor analysis. The precipitated cells were resuspended with 1 ml red blood cell lysis buffer. H&E staining was performed on each suspension smear, and cell classification and count were performed. Neutrophils, macrophages and lymphocytes were counted under an optical microscope using standard morphological standards.

### Histological Examination

The left lung was fixed with 10% paraformaldehyde for 24 h, the excess tissues were removed and embedded in paraffin. Lung sections were prepared (4 µm), hematoxylin -eosin (H&E) staining (Zsbio, China) for histological examination.

### ELISA Detection

The supernatant of BALF was used to detect the concentration of inflammatory factors including IL-1α, IFN-γ, IL-6, TNF-α and IL-4 using enzyme-linked immunosorbent assay (ELISA) kits (Jianglai biotech, shanghai, China) in accordance with the manufacturer’s protocol.

### Statistical Analysis

Statistical analysis was performed using GraphPad Prism 7.0 software. Differences between experimental and control group were assessed by Student’s *t* test. Significant differences among multiple groups were detected by one-way ANOVA. *P* <0.05 was considered as statistically significance, *P <0.05, **P <0.01, ***P <0.001, NS: nonsignificant.

## Results

### Molecular Docking

We docked the 15 natural compounds and 17 chemical compounds to the crystal structure of SARS-CoV-2 M^pro^. The 2D structures of the 15 natural compounds and the corresponding Glide XP docking scores are listed in Table 1 and the 2D structures of the 17 chemical compounds and the corresponding Glide XP docking scores are listed in Supplementary Table S1. Among them, four compounds (i.e. Myricetin, Vitexin, Genistin and Oleuropein) show docking scores lower than −8.0, which indicates that these compounds might have effective inhibition on SARS-CoV-2 M^pro^ activity.

**TABLE 1:** List of drug molecular docking and primary FRET assay against SARS-CoV-2 M^pro^.

Flavonoids compounds					
			
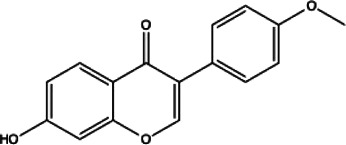	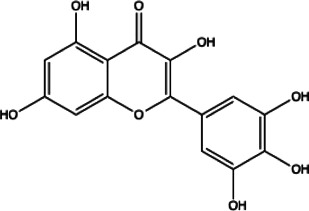	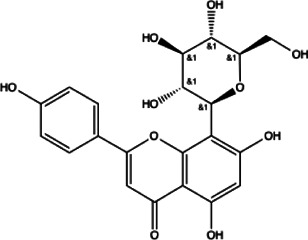	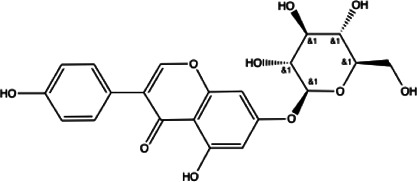
Formononetin(1)	Myricetin(2)	Vitexin(3)		Genistin(4)
docking score (−5.988)	docking score (−8.473)	docking score (−8.359)		docking score (−7.934)	
			
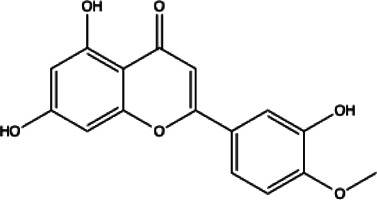	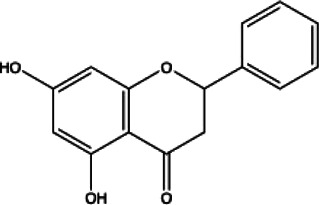	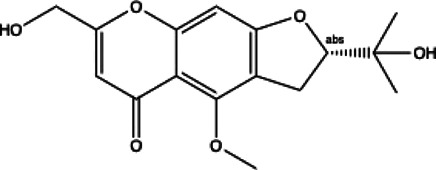
Diosmetin(5)	Pinocembrin(6)	Cimifugin(7)		
docking score (−7.761)	docking score (−6.748)	docking score (−6.578)		
**Coumarins compounds**					
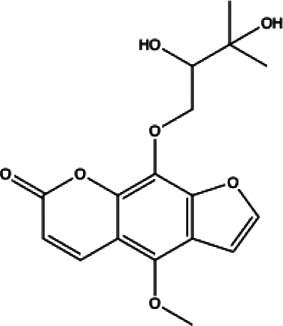	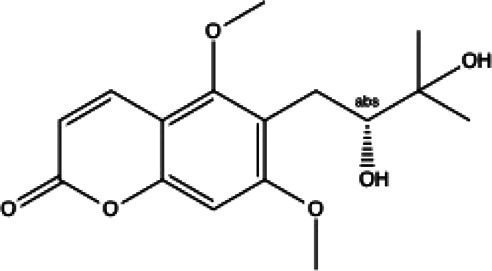	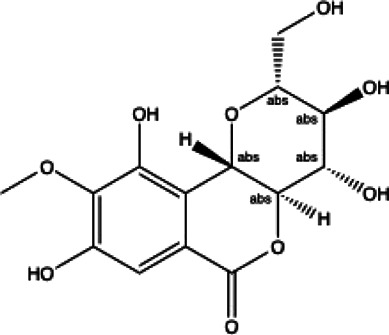
			
Byakangelicin(8)	Toddalolactone(9)	Bengenin(10)		
docking score (−7.177)	docking score (−6.527)	docking score (−6.143)		
**Terpenoid**					
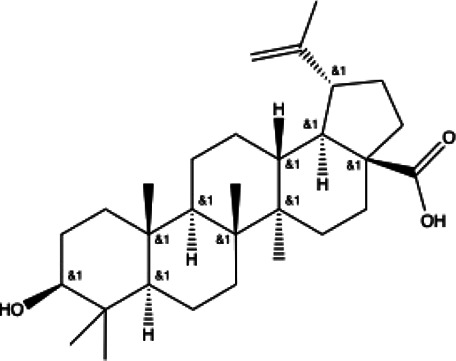	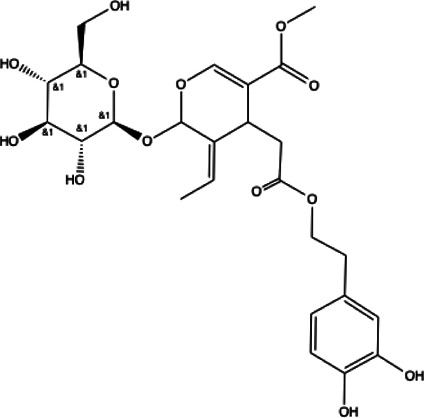
	
Betulinic acid(11)	Oleuropein(12)
docking score (−3.623)	docking score (−10.33)
**Henolic compound**	**Aldehyde compound**	**Steroid**
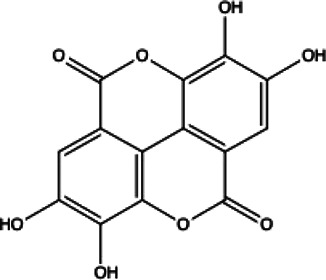	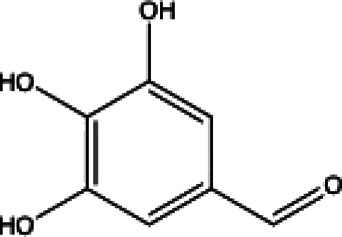	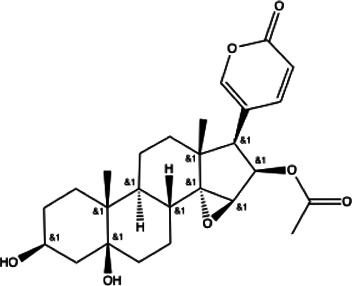
Ellagic acid(13) docking score (−7.222)	3,4,5-Trihydroxybenzaldehyde(14) docking score (−3.828)		Cinobufotalin(15) docking score (−4.24)	

### Fluorescence Resonance Energy Transfer (FRET)-Based Screening Assay

**FIGURE 1:**
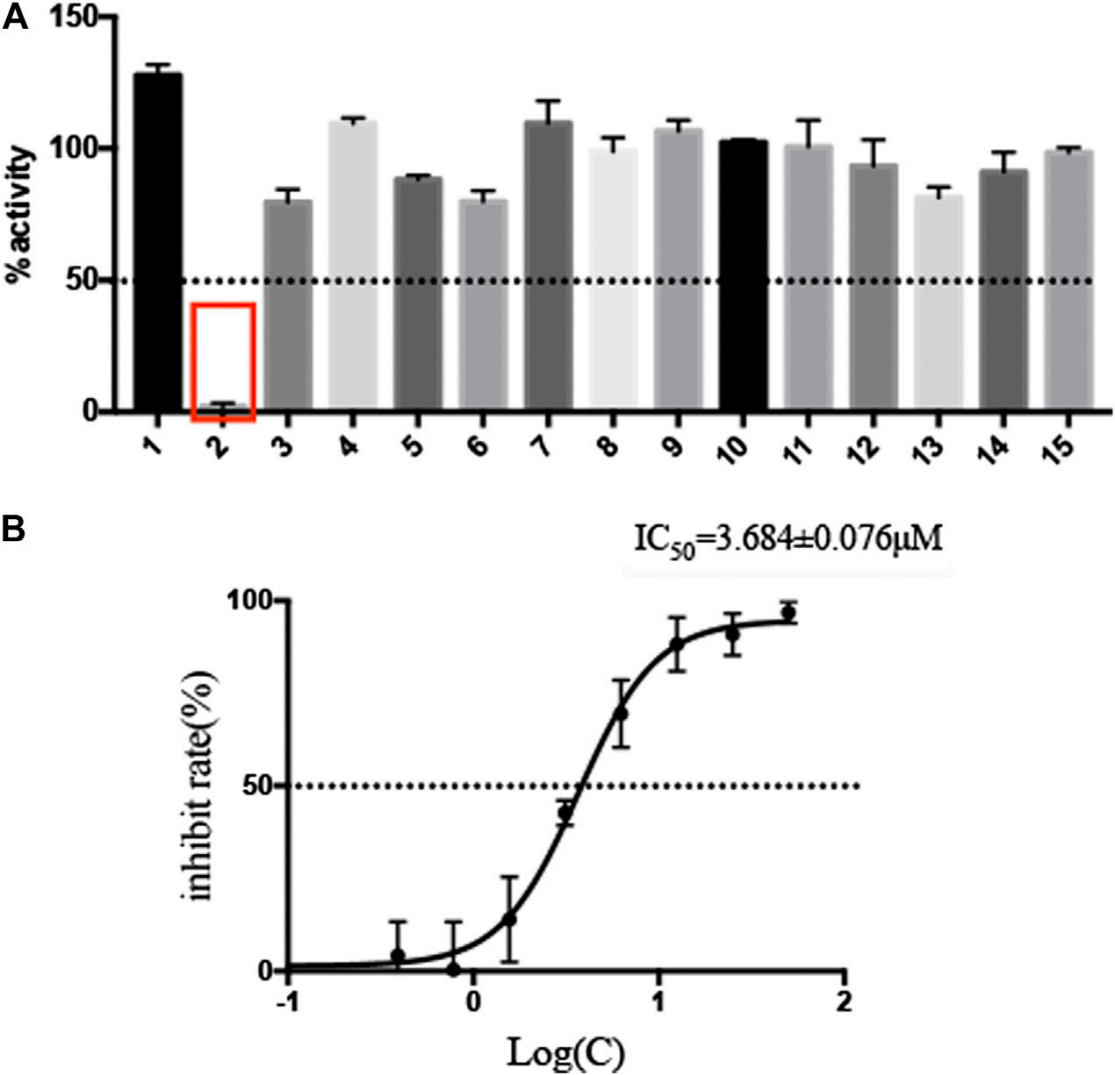
Screening of natural compounds against SARS-CoV-2 M^pro^ and the inhibitory activity of Myricetin *in vitro*. **(A)**. 50 μM compound was pre-incubated with 0.3 μM SARS-CoV-2 M^pro^ at 37°C for 10 min, and then 20 μM FRET substrate was added to the reaction mixture to initiate the reaction. The excitation wavelength is 340 nm and the emission wavelength is 490 nm for fluorescence measurement. Results Inhibition rate (%) = (RFU100% enzyme activity control-RFU sample)/(RFU100% enzyme activity control-RFU blank control) × 100%. The results are average ± standard deviation of three repeats. **(B)** The inhibitory assay of Myricetin show efficient inhibition for M^pro^. Error bars: mean ± S.D. of three independent replicates.

The selected 15 natural compounds belong to 6 different categories, 7 compounds are flavonoids, 3 compounds are coumarins. 2 compounds is terpenoid, one is henolic, one is aldehyde and one is steroid. We screened these 15 natural compounds and 17 chemical compounds by fluorescence resonance energy transfer enzymatic assay at a final concentration of 50 µM (Figure 1A and Supplementary Figure S3). We identified that Myricetin has effective inhibition on enzymatic activity, the inhibition rate reached 97.79%, but, other compounds did not show obvious inhibitory activity, including Oleuropein, Vitexin and Genistin with low molecular docking scores.

### Myricetin Inhibit the SARS-CoV-2 M^pro^ Activity and Its Structural Basis

**FIGURE 2:**
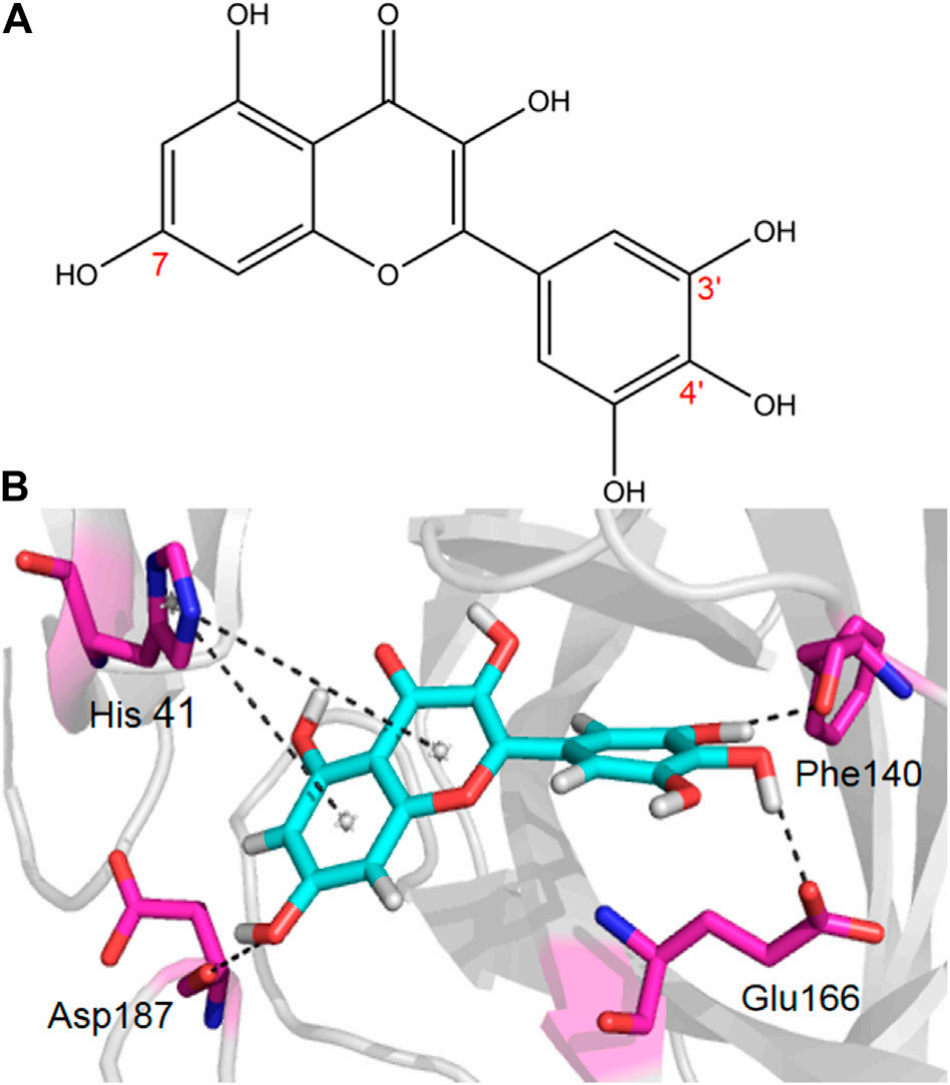
The binding mode of Myricetin in SARS-CoV-2 Mpro. Hydrogen bonds and *π*-π interactions between Myricetin and SARS-CoV-2 M^pro^ are represented by dashed lines.

Given the encouraging results from the primary screening, we then further characterized the inhibitory activity of Myricetin in a dose gradient and the Myricetin inhibited SARS-CoV-2 M^pro^ with 50% inhibitory concentration values (IC_50_) of 3.684 ± 0.076 μM (Figure 1B). As the positive control, Ebselen inhibited M^pro^ with IC_50_ of 0.5417 ± 0.0306 μM (Supplementary Figure S4). We measured the cell toxicity of Myricetin to BEAS-2B cell, after treated with Myricetin for 48 h, Myricetin had no cytotoxicity within 50 μM (Supplementary Figure S2). We also identified the structural basis of Myricetin and M^pro^. To investigate the stability of Myricetin inside the active site of SARS-CoV-2 M^pro^, we performed 100 ns MD simulation on the binding complex of SARS-CoV-2 M^pro^ with Myricetin. The revealed binding mode of Myricetin with SARS-CoV-2 M^pro^ is depicted in Figure 2, and the interaction details between Myricetin and SARS-CoV-2 M^pro^ over time are shown in Supplementary Figure S5. The calculated RMSD shows the stability of the system (Supplementary Figure S5A). RMSF shows fluctuations are at the N-terminal and C-terminal ends of the protein (Supplementary Figure S5B). The chromone ring of Myricetin interacts with the imidazole side chain of His41 through *π*-π stacking (with the centroids distance of ∼6.1 Å, Supplementary Figure S5C). The 3’− and 4’−hydroxyl of Myricetin form hydrogen bonds with the backbone oxygen of Phe140 and the side chain carboxyl oxygen of Glu166 (with the hydrogen bond lengths of ∼2.3 Å and ∼2.0 Å, Supplementary Figures S5D,E). The 7-hydroxyl of Myricetin forms a hydrogen bond with the backbone oxygen of Asp187 (with the hydrogen bond length of ∼2.4 Å, Supplementary Figure S5F). These values indicate that Myricetin maintains its position in the binding pocket of SARS-CoV-2 M^pro^. The binding free energy of Myricetin with SARS-CoV-2 Mpro is −32.98 kcal/mol calculated using the MMGBSA method. We also performed docking and MD simulation for the control drug Ebselen in the same binding pocket of Myricetin (i.e. the Cys145 site). The binding pose of Ebselen in the Cys145 pocket is shown in Supplementary Figure S6A. The benzisoselenazolone ring and the benzene ring of Ebselen interact with the imidazole side chain of His41 through *π*-π stacking (with the centroids distance of ∼4.8 and ∼5.4 Å, Supplementary Figures S6B,C) in this pocket, and the binding free energy is only −17.68 kcal/mol. Fortunately, we found that the crystal structure of Ebselen bound to SARS-CoV-2 M^pro^ was just deposited in the protein data bank quite recently (PDBID: 7BFB). In this structure, Ebselens covalently bind to SARS-CoV-2 M^pro^ through four binding sites, e.g. the Cys44 site, the Cys145 site, the Cys156 site and the Cys300 site. The aforementioned binding mode (in Supplementary Figure S6A) only reflects the binding of Ebselen in the Cys145 site. The molecular size of Ebselen is small, it can easily reach these four Cys sites in SARS-CoV-2 M^pro^. Moreover, the sulfydryl group of Cys is quite active, when Ebselen enters the active site, they will react quickly and form the covalent complex. These provide the structural basis for Ebselen having lower IC_50_ than other ligands.

### Myricetin Reduced the Inflammatory Response in Bleomycin-Treated Mice and Macrophage

**FIGURE 3:**
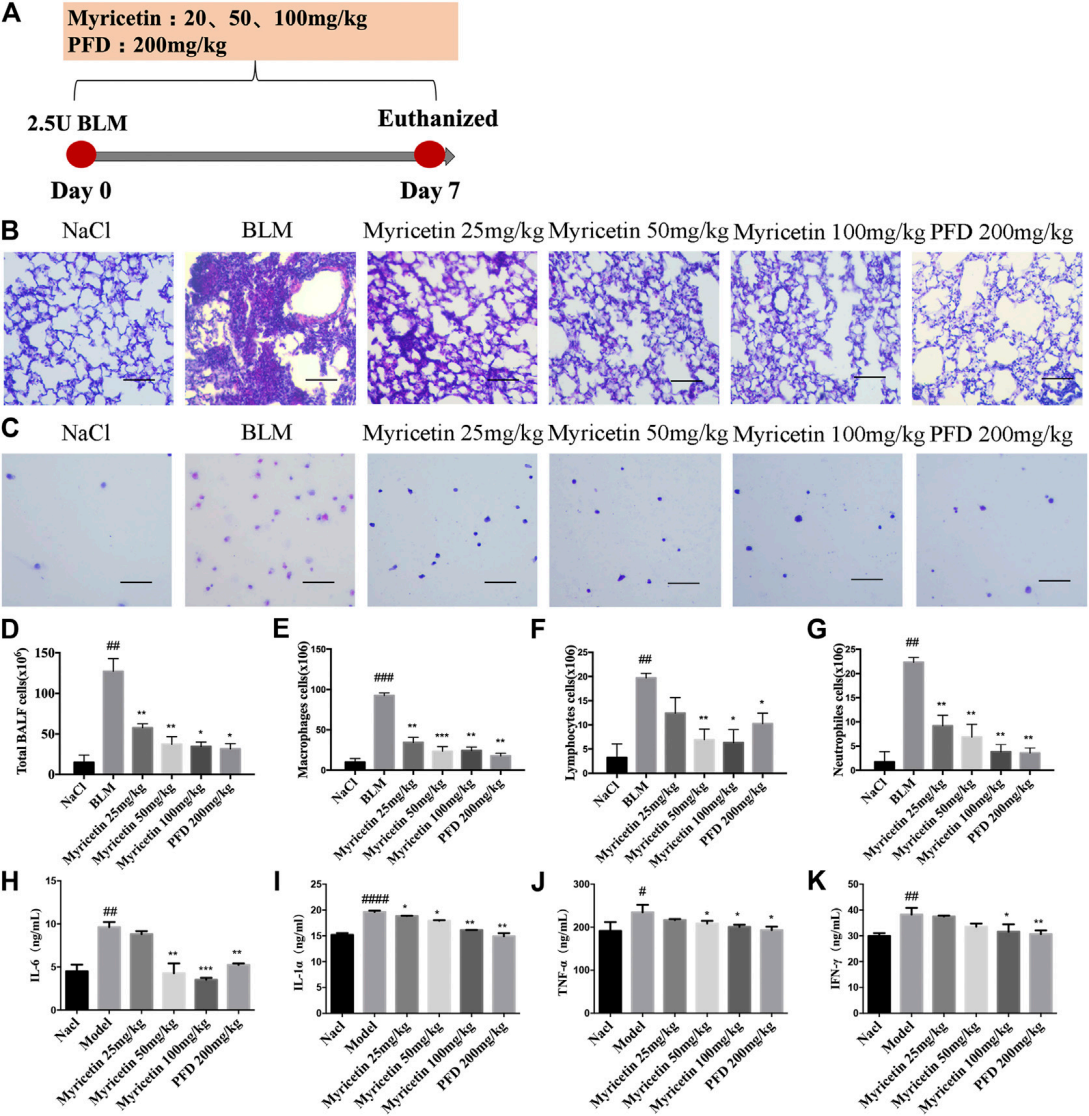
Myricetin reduces the inflammatory response in BLM-treated mice. **(A)** Dosing regimen in BLM-induced inflammatory model. **(B–C)** H&E staining of left lung tissues (B, Scale: 50 μm) and inflammatory cells in BALF (C, Scale: 20 μm) of each group. **(D)** Total number of cells from BALF in each group. **(E)** Counts of macrophages in BALF. **(F)** Counts of lymphocytes in BALF. **(G)** Counts of Neutrophiles in BALF. **(H–K)** The expression of inflammatory factors including IL-6, IL-1α, TNF-α and IFN-γ in BALF were detected by ELISA. Data are shown as mean ± SD. # represent the difference between NaCl and BLM-treated group, ##*P* < 0.01, ###*P* < 0.001, ####*P* < 0.0001. * represent the difference between BLM-treated and treatment group, *P < 0.05, **P < 0.01, ***P < 0.001, ****P < 0.0001.

To study the anti-inflammatory effect of Myricetin on lung injury, a BLM-induced lung injury model was established. The drug was administered continuously for 7 days, and pirfenidone was used as a positive control (Figure 3A). The results of H&E staining in lung biopsy showed that Myricetin significantly improved the infiltration of inflammatory cells in BLM damaged lung tissue (Figure 3B). In the BALF of BLM-treated mice, the total number of inflammatory cells and the number of different inflammatory cells were significantly up-regulated, while the number of inflammatory cells in Myricetin-treated mice was significantly down-regulated in a dose dependent manner. The effect of high dose Myricetin (100 mg/kg) is similar to that of the positive drug pirfenidone (Figures 3C–G). In addition, the expression levels of inflammatory factors such as IL-6, TNF-α,IFN-γ and IL-1α in BALF were measured, and the results showed that Myricetin significantly inhibited the expression levels of inflammatory factors (Figures 3H–K). These data showed that Myricetin reduced lung inflammation in BLM-induced mice.

## Discussion

The popularity of coronavirus disease 2019 (COVID-19) has given rise to an urgent need for new therapy strategies (Wu et al., 2020). At present, there is no available specific drugs targeting SARS-CoV-2 (Luo et al., 2020), but new drug candidates targeting the SARS-CoV-2 M^pro^ to inhibit the viral replication are being explored with the X-ray crystal structure was reported (Garg and Roy, 2020; Jin et al., 2020; Ma et al., 2020; Zhang et al., 2020). Now, a large number of compounds have been screened by structure-based virtual screening, including FDA approved drug libraries (Kandeel and Al-Nazawi, 2020), drug candidates in clinical trials (Mahanta et al., 2020) and other pharmacologically active compounds (Vijayakumar et al., 2020). Lopinavir and nelfinavir (Costanzo et al., 2020; Reiner et al., 2020), the FDA approved antiretroviral drug used against HIV, showed excellent binding affinity with the M^pro^ through virtual screening and *in silico* studies. However, it were proved that they have no inhibitory activity at 20 μM by FRET-based assay (Hung et al., 2020), which reflecting the fact that no benefit was observed in patients with severe COVID-19. Tens of thousands of phytochemicals and Chinese medicinal agents, such as flavonoids, garlic, naturally occurring coumarin derivatives and green tea polyphenols, have been determined to have higher affinity than some marketed drugs and may be promising candidates, but their usefulness for targeting M^pro^ needs experimental validation and clinical manifestation (Ghosh et al., 2020; Joshi et al., 2020; Li et al., 2020).

We compared the binding affinity of 15 natural compounds, contain of flavonoids, coumarins, terpenoids, henolic, aldehyde and steriod compounds, with SARS-CoV-2 M^pro^ through virtual analysis. Oleuropein, Myricetin and vitexin have high affinity with M^pro^, however, only Myricetin exhibit significant inhibition with IC_50_ 3.684 ± 0.076 μM by FRET-based assay. Structurally, Myricetin can interact with His41 through *π*-π stacking and form hydrogen bonds with Phe140, Glu166 and Asp187 in the catalytic center of SARS-CoV-2 M^pro^. This result indicates that the anti-viral activity test based on experiments are necessary for developing more effective and reliable anti-SARS-CoV-2 drugs.

COVID-19 is an inflammatory disease caused by SARS-CoV-2 (Zhu et al., 2020). Excessive inflammation is central to a poor prognosis, and associated with inflammatory mediators such as IL-6 and lactate dehydrogenase (LDH) (Conti et al., 2020; Rodrigues et al., 2021). Here, we further evaluated the effect of Myricetin on pulmonary inflammation with bleomycin treated mice. The results showed that Myricetin can effective inhibit the infiltration of inflammatory cells and the secretion of inflammatory factors in the lung, especially lymphocytes and IL-6.

## Conclusion

In a word, Myricetin may be an potential candidate drug for COVID-19 therapy by both anti-SARS-CoV-2 and anti-inflammation. Small-molecule bioactive natural products could be a useful source of SARS-CoV-2 M^pro^ inhibitors and an effective first line of defense against COVID-19.

## Data Availability

The datasets presented in this study can be found in online repositories. The names of the repository/repositories and accession numbers can be found in the article/Supplementary Material.
