# Supplementary material for: Myricetin Inhibits SARS-CoV-2 Viral Replication by Targeting Mpro and Ameliorates Pulmonary Inflammation
Source: Front Pharmacol. 2021 Jun 17;12:669642. doi: 10.3389/fphar.2021.669642 (PMC8248548; doi:10.3389/fphar.2021.669642)
Supplement: Supplementary file 1 [file DataSheet1.docx]

**Fig. S1 Myricetin reduces the expression of IL-4 in BLM-treated mice.** The expression of IL-4 in BALF was detected by ELISA. Data are shown as mean ± SD. # represent the difference between NaCl and BLM-treated group, ##P<0.01, ###P< 0.001, ####P< 0.0001. * represent the difference between BLM-treated and treatment group, *P < 0.05, **P < 0.01, ***P < 0.001, ****P < 0.0001.

**Fig. S2 The cell toxicity of Myricetin to BEAS-2B cell.** The BEAS-2B cell was treated with various concentrations of myricetin for 48h and the cell toxicity of myricetin was measured by MTT assay. Three experimental results were repeated at least three times.

**Fig. S3 Screening of marketed drugs against SAS-CoV-2 M^pro^ in *vitro*.** 50 μM compound was pre-incubated with 0.3μM SARS-CoV-2 M^pro^ at 37°C for 10 minutes, and then 20 μM FRET substrate was added to the reaction mixture to initiate the reaction. The excitation wavelength is 340nm and the emission wavelength is 490nm for fluorescence measurement. Results Inhibition rate (%) = (RFU100% enzyme activity control-RFU sample) / (RFU100% enzyme activity control-RFU blank control) × 100%. The results are average ±standard deviation of three repeats.

| **Table S1.** List of chemical compounds for molecular docking. |
| --- |
| 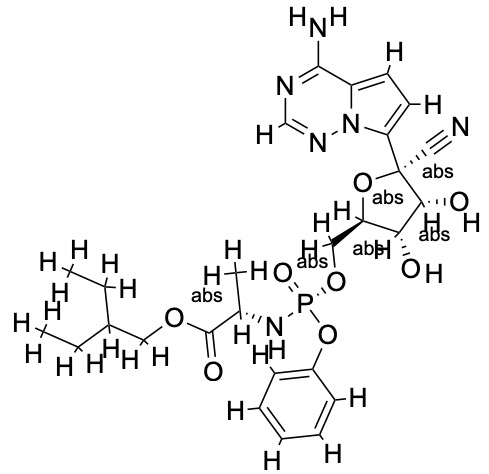 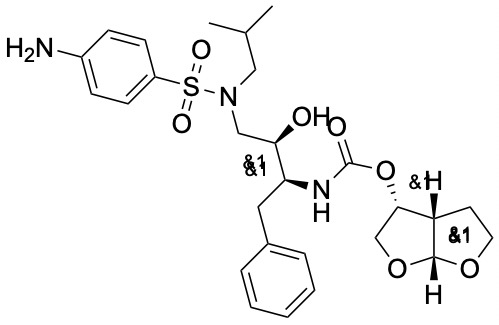 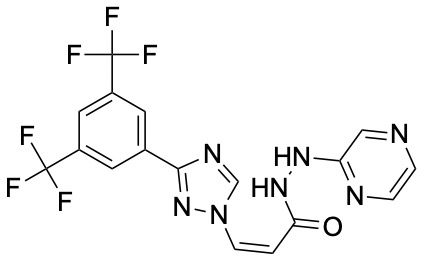  Remdesivir Darunavir Selinexor  docking score(-7.602) docking score(-7.388) docking score(-6.328)  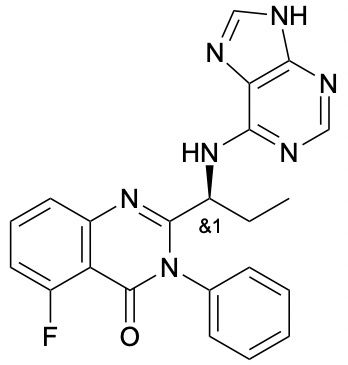 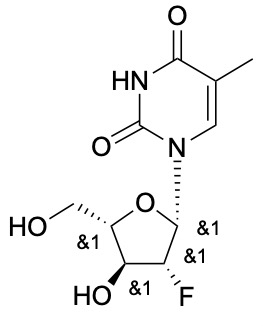 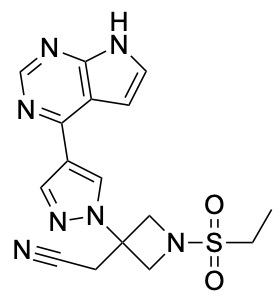  Idelalisib Clevudine Baricitinib  docking score(-5.976) docking score(-5.733) docking score(-5.491)    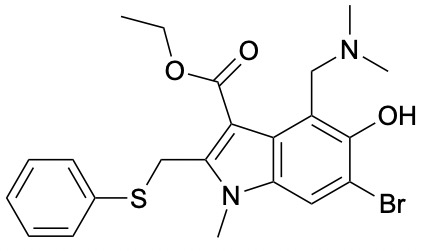 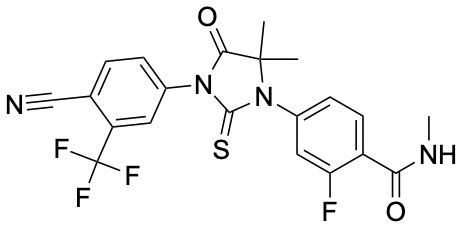 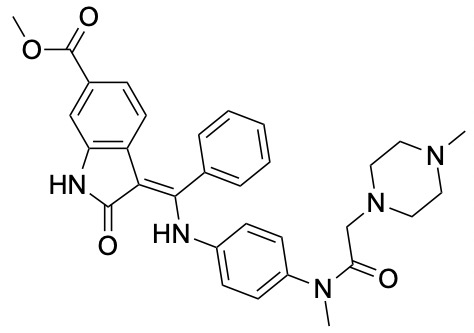  Arbidol Enzalutamide Nintedanib  docking score(-5.485) docking score(-5.368) docking score(-5.256)  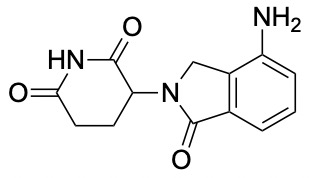 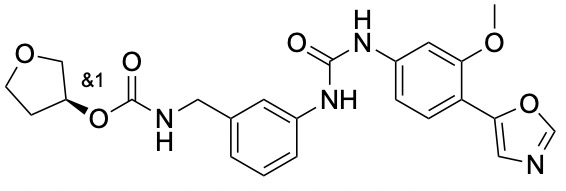 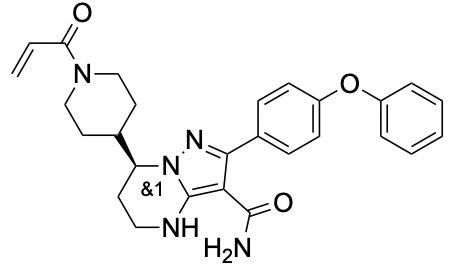  Lenalidomide Merimepodib Zanubrutinib  docking score(-5.217) docking score(-5.066) docking score(-5.061) 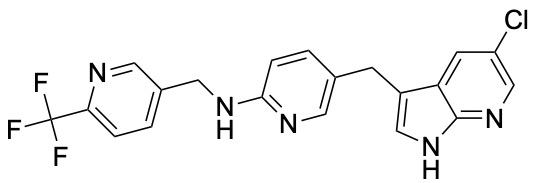 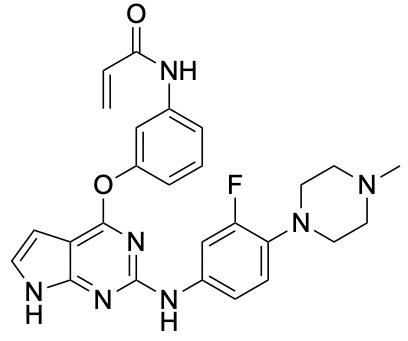 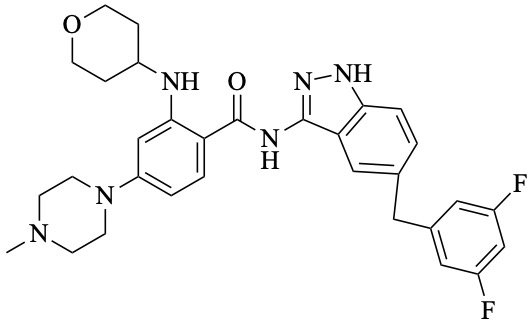  Pexidartinib Avitinib Entrectinib  docking score(-4.874) docking score(-4.59) docking score(-4.218)  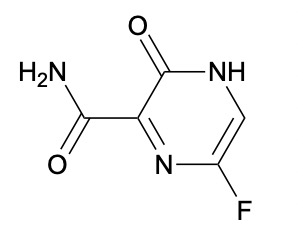 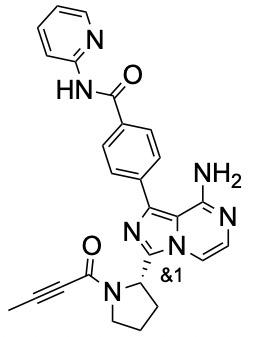  Favipiravir Acalabruinib  docking score(-4.11) docking score(-3.904) |

**Fig. S4 The inhibitory activity of Ebselen agaist SAS-CoV-2 M^pro^ in *vitro*.** The inhibitory assay of Ebselen show efficient inhibition for M^pro^. Error bars: mean ± S.D. of three independent replicates.

**
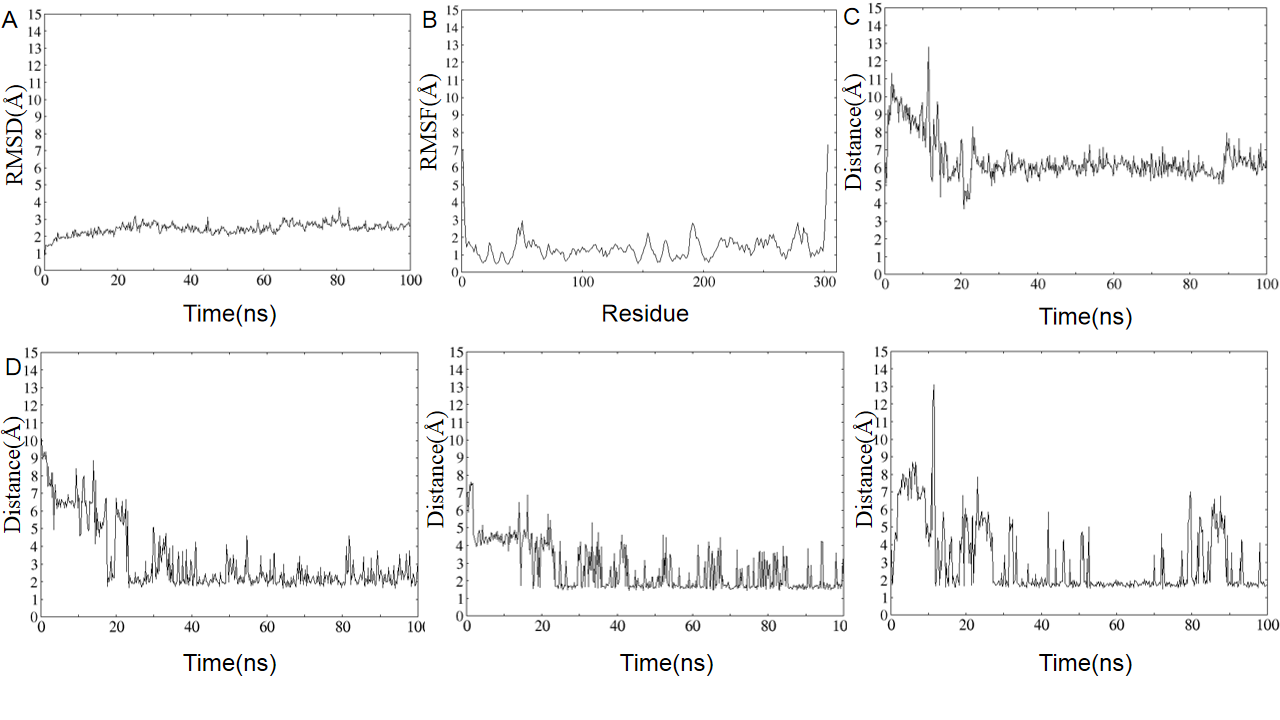
**

**Fig. S5 Interaction details between Myricetin and SARS-CoV-2 M^pro^ during the 100-ns MD simulation.** (A) RMSD, (B) RMSF, (C) centroids distance between the chromone ring of Myricetin and the imidazole of His41, (D) hydrogen bond length between the 3’-hydroxyl of Myricetin and the backbone oxygen of Phe140, (E) hydrogen bond length between the 4’-hydroxyl of Myricetin and the side chain carboxyl oxygen of Glu166, (F) hydrogen bond length between the 7-hydroxyl of Myricetin and the backbone oxygen of Asp187.


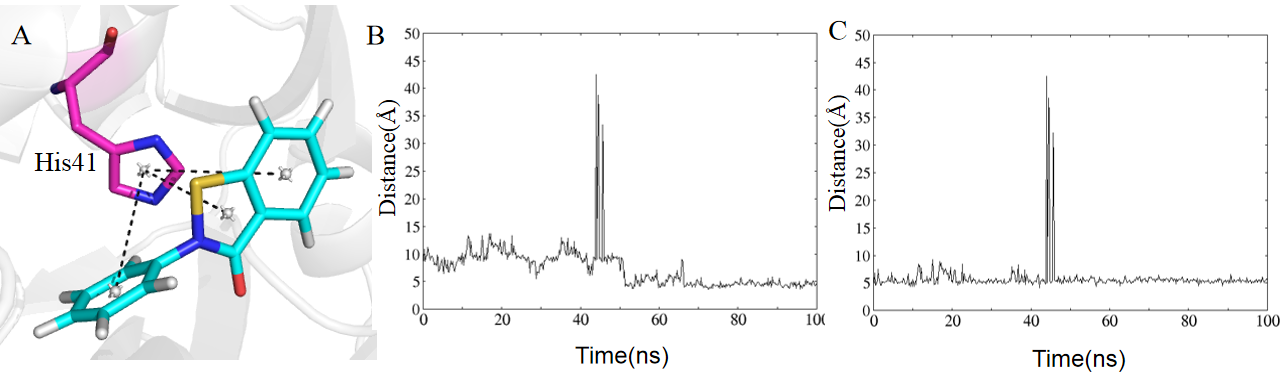


**Fig. S6 Interaction details between Ebselen and SARS-CoV-2 M^pro^ in the Cys145 site during the 100-ns MD simulation.** (A) binding mode, (B) centroids distance between the benzisoselenazolone ring of Ebselen and the imidazole of His41, (C) centroids distance between the benzene ring of Ebselen and the imidazole of His41.
